# “Rosary Sign” at Somatostatin Receptor PET in a Case of Recurrent Meningioma

**DOI:** 10.3390/diagnostics14222608

**Published:** 2024-11-20

**Authors:** Cesare Michele Iacovitti, Davide Giovanni Bosetti, Barbara Muoio, Marco Cuzzocrea, Gaetano Paone, Giorgio Treglia

**Affiliations:** 1 Division of Nuclear Medicine, Imaging Institute of Southern Switzerland, Ente Ospedaliero Cantonale, 6500 Bellinzona, Switzerland; cesaremichele.iacovitti@eoc.ch (C.M.I.); marco.cuzzocrea@eoc.ch (M.C.); gaetano.paone@eoc.ch (G.P.); 2 Division of Radiation Oncology, Oncology Institute of Southern Switzerland, Ente Ospedaliero Cantonale, 6500 Bellinzona, Switzerland; davidegiovanni.bosetti@eoc.ch; 3 Division of Medical Oncology, Oncology Institute of Southern Switzerland, Ente Ospedaliero Cantonale, 6500 Bellinzona, Switzerland; barbara.muoio@eoc.ch; 4 Faculty of Biomedical Sciences, Università della Svizzera Italiana, 6900 Lugano, Switzerland; 5 Faculty of Biology and Medicine, University of Lausanne, 1015 Lausanne, Switzerland

**Keywords:** meningioma, somatostatin receptor, PET/CT, PRRT, rosary sign, brain tumors, nuclear medicine, hybrid imaging

## Abstract

We present the case of a 60-year-old male with recurrent atypical meningioma in the right parietal lobe, previously treated with surgery and radiation therapy. Magnetic resonance imaging (MRI) performed 5 years after radiation therapy suggested a possible recurrence. A somatostatin receptor positron emission tomography/computed tomography (SR-PET/CT) scan with Gallium-68 DOTATATE was performed to confirm this suspicion. SR-PET/CT confirmed the presence of recurrent meningioma, showing a novel “rosary sign” with multiple adjacent areas of focal tracer uptake along the resection margins of the previous surgical site in the right parietal region. This novel imaging pattern improved diagnostic accuracy by detailing disease extent and identifying additional lesions not visible via MRI. Given the failure of prior treatments and high SR expression, peptide receptor radionuclide therapy (PRRT) was proposed as a therapeutic option for the patient.

**Figure 1:**
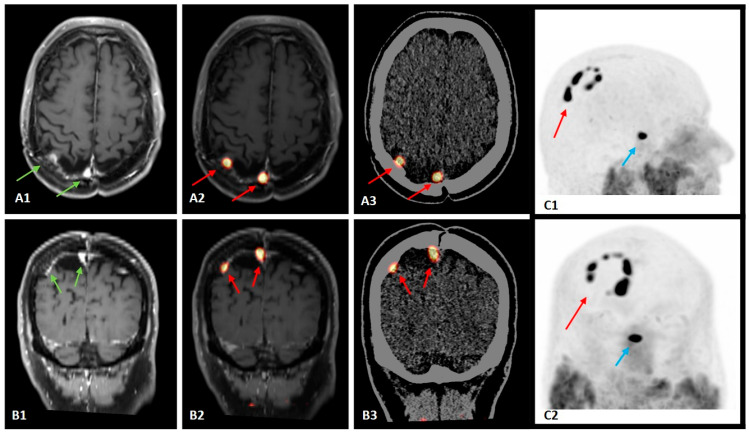
A 60-year-old male with recurrent atypical meningioma, previously treated with surgery (6 years before) and radiation therapy for a local recurrence (5 years before), underwent segmental brain somatostatin receptor positron emission tomography/computed tomography (SR-PET/CT) for restaging after magnetic resonance imaging (MRI) findings suggested a possible recurrence. SR-PET/CT was performed 60 min after the injection of 200 MBq of Gallium-68 DOTATATE (a radiolabeled somatostatin analogue), and PET images were also fused with the recent MRI images. SR-PET image analysis was performed by using qualitative criteria: areas of increased radiopharmaceutical uptake compared to the background, excluding the sites of physiological radiotracer uptake, were considered abnormal. Furthermore, semi-quantitative PET image analysis was performed by using the maximal standardized uptake value (SUV_max_). Axial and coronal T1-weighted MRI images (**A1**,**B1**), fused PET/MRI images (**A2**,**B2**), fused PET/CT images (**A3**,**B3**) and maximum intensity projection PET images (lateral view (**C1**), anterior view (**C2**)) revealed a novel “rosary sign” with multiple adjacent areas of focal abnormal radiopharmaceutical uptake (red arrows) along the resection margins of the previous surgical site in the right parietal region, corresponding to hyperintense lesions at T1-weighted MRI (green arrows), while the pituitary showed physiological tracer uptake (blue arrows). The “rosary sign” was particularly relevant because it highlighted multiple foci of abnormal radiopharmaceutical uptake linked together, suggesting disease recurrence and confirming the suspicion raised by MRI, as well as identifying a higher number of lesions. The highest SUV_max_ of the meningiomatous lesions was 16.9. Due to the high SR expression demonstrated by SR-PET/CT with this novel imaging pattern and considering progressing/recurrent disease after multiple treatments, this patient was addressed with peptide receptor radionuclide therapy (PRRT) [1,2,3]. SR-PET/CT is indicated for the differential diagnosis of brain lesions suspicious for meningiomas, delineation of meningioma extent, detection multifocal disease/extracranial metastases, and monitoring of disease progression or diagnosis of recurrence, playing a crucial role in these clinical assessments [1,2,4,5,6,7,8,9]. PRRT is a well-tolerated treatment option for both meningiomas and neuroendocrine tumors with high somatostatin receptor expression. Patients with progressive/recurrent/treatment refractory meningiomas and increased SR expression are ideal candidates for PRRT if a previous SR-PET/CT demonstrates high SR expression [1,2,3].

In the interesting described case with recurrent meningioma, SR-PET enabled the accurate evaluation of the meningioma recurrence based on its increased SR expression, with a novel uptake pattern (“rosary sign”) suggesting the use of PRRT as a further therapeutic step.

## Data Availability

The data presented in this article are available on request from the corresponding author.

## References

[1] Albert N.L., Preusser M., Traub-Weidinger T., Tolboom N., Law I., Palmer J.D., Guedj E., Furtner J., Fraioli F., Huang R.Y. (2024). Joint EANM/EANO/RANO/SNMMI practice guideline/procedure standards for diagnostics and therapy (theranostics) of meningiomas using radiolabeled somatostatin receptor ligands: Version 1.0. Eur. J. Nucl. Med. Mol. Imaging.

[2] Filippi L., Palumbo I., Bagni O., Schillaci O., Aristei C., Palumbo B. (2022). Somatostatin Receptor Targeted PET-Imaging for Diagnosis, Radiotherapy Planning and Theranostics of Meningiomas: A Systematic Review of the Literature. Diagnostics.

[3] Mirian C., Duun-Henriksen A.K., Maier A., Pedersen M.M., Jensen L.R., Bashir A., Graillon T., Hrachova M., Bota D., van Essen M. (2021). Somatostatin Receptor-Targeted Radiopeptide Therapy in Treatment-Refractory Meningioma: Individual Patient Data Meta-analysis. J. Nucl. Med..

[4] Palmisciano P., Watanabe G., Conching A., Ogasawara C., Ferini G., Bin-Alamer O., Haider A.S., Sabini M.G., Cuttone G., Cosentino S. (2022). The Role of [⁶⁸Ga]Ga-DOTA-SSTR PET Radiotracers in Brain Tumors: A Systematic Review of the Literature and Ongoing Clinical Trials. Cancers.

[5] Treglia G., Raditchkova M., Giovanella L., Stelmes J.J., Bosetti D.G., Martucci F. (2021). Two Birds with One Stone: Skull Base Meningioma and Jugulotympanic Paragangliomas with Somatostatin Receptor Positron Emission Tomography. Diagnostics.

[6] Helgebostad R., Revheim M.E., Johnsrud K., Amlie K., Alavi A., Connelly J.P. (2022). Clinical Applications of Somatostatin Receptor (Agonist) PET Tracers beyond Neuroendocrine Tumors. Diagnostics.

[7] Muoio B., Espeli V., Treglia G. (2023). Neuro-Oncology and Positron Emission Tomography: “Just Can’t Get Enough”. Cancers.

[8] Albano D., Treglia G., Dondi F., Bertagna F. (2022). Prevalence of Brain Incidental Lesions Detected by ^68^Ga-DOTA Peptides PET/CT. Medicina.

[9] Treglia G., Muoio B., Trevisi G., Mattoli M.V., Albano D., Bertagna F., Giovanella L. (2019). Diagnostic Performance and Prognostic Value of PET/CT with Different Tracers for Brain Tumors: A Systematic Review of Published Meta-Analyses. Int. J. Mol. Sci..

